# Supplementary material for: Tree height strongly affects estimates of water-use efficiency responses to climate and CO2 using isotopes
Source: Nat Commun. 2017 Aug 18;8:288. doi: 10.1038/s41467-017-00225-z (PMC5561090; doi:10.1038/s41467-017-00225-z)
Supplement: Supplementary file 1 — Supplementary Information [file 41467_2017_225_MOESM1_ESM.pdf]

File name: Supplementary Information
Description: Supplementary Figures, Supplementary Table and Supplementary References

File name: Peer Review File
Description:

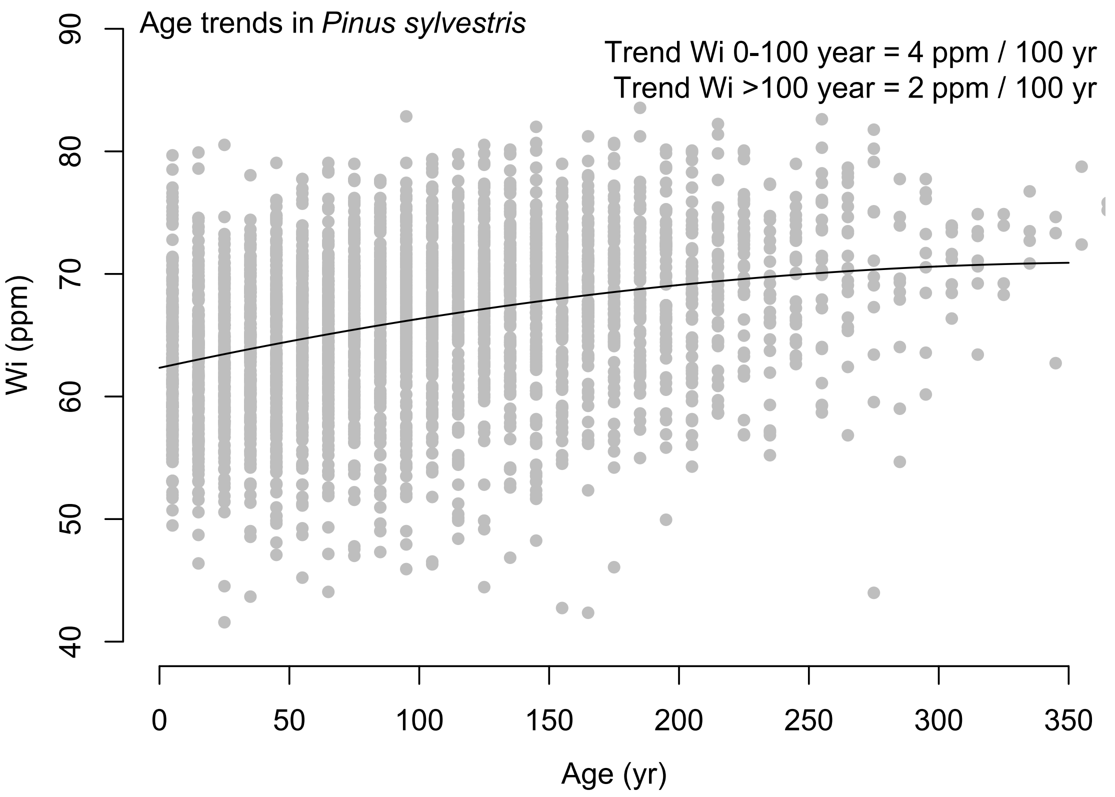


**Supplementary Figure 1. Age trends in $W_i$ in subfossil trees of *Pinus sylvestris* from Finland.** Age trends before and after reaching 100 years of age are both significant (P< 0.000), but trends after reaching 100 years are much weaker (2 ‰ /100 years) compared to the trends during the first 100 years of a trees' life. $W_i$ is estimated using $\delta^{13}C$ data from 10-year ring sections from the dataset of Helama *et al.*[1]. For further details see Methods.

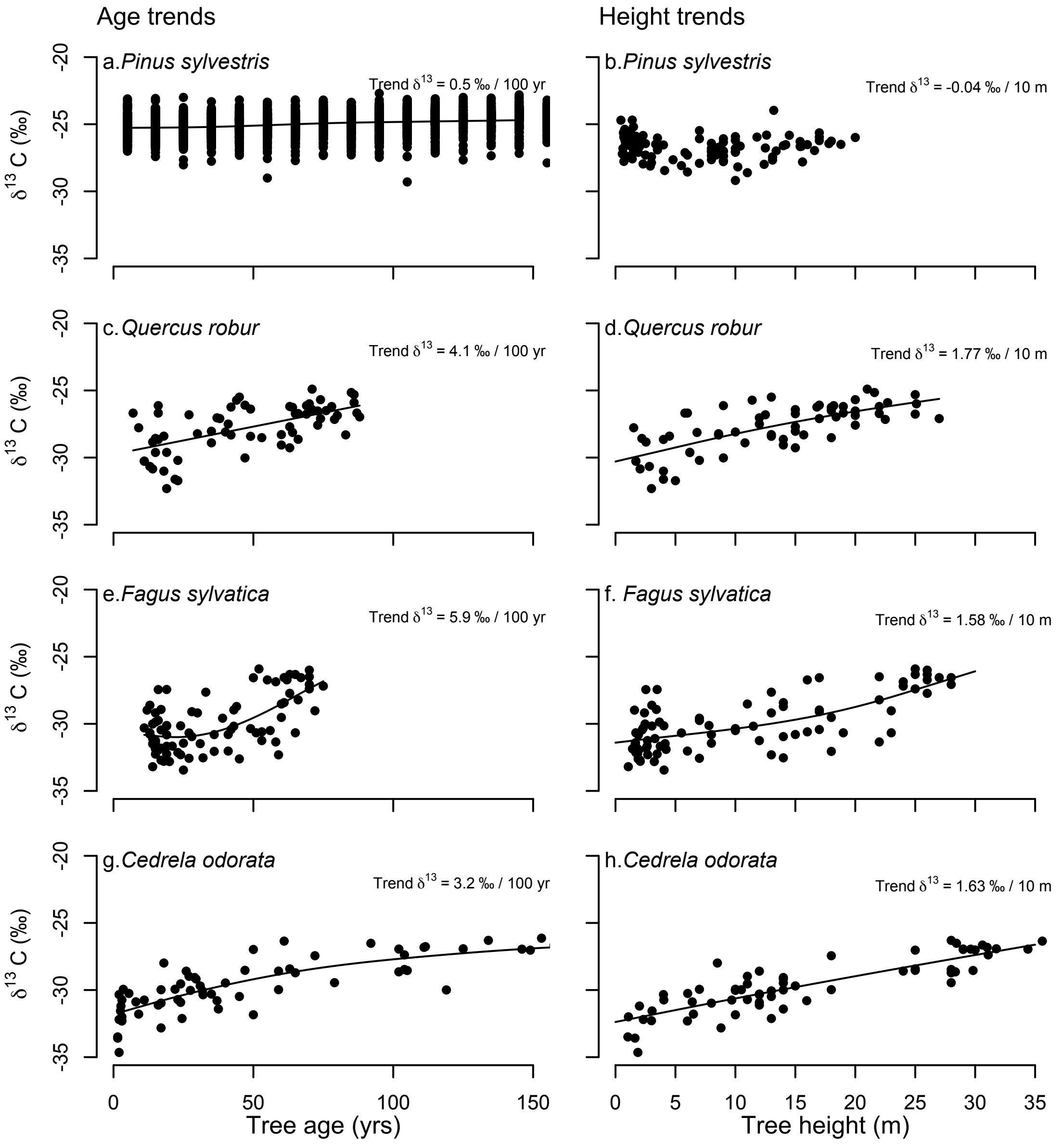


**Supplementary Figure 2. Raw δ$^{13}$C values plotted versus tree age and tree height for the four species.** δ$^{13}$C in all panels, except for panel a, represent the average δ$^{13}$C of the last 5 rings (see Methods). Age trends in *Pinus* in panel a are estimated using δ$^{13}$C of 10-year ring sections of subfossil trees from Northern Finland[1]. Curve estimates were performed using General Additive Mixed Models (GAMM) from the gamm4 R package[2].

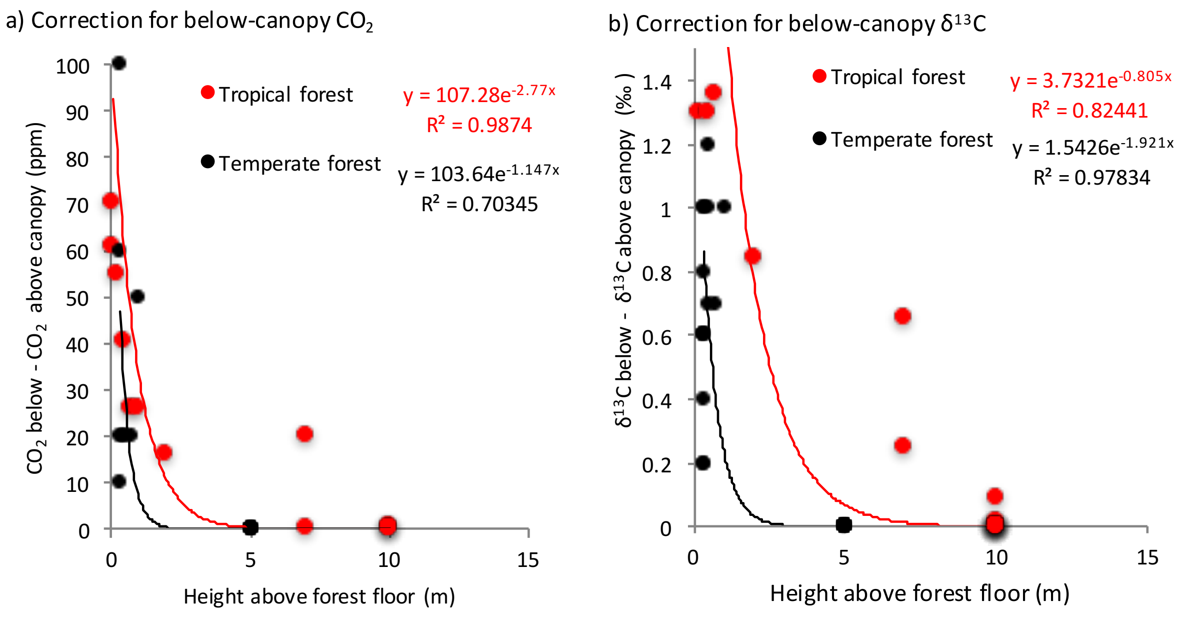


**Supplementary Figure 3 Estimation of below-canopy profiles in $CO_2$-concentration and $\delta^{13}C_{air}$ for tropical and temperate forests using literature data.** Graphs show the differences in $CO_2$ concentration (panel a), and $\delta^{13}C$ of the $CO_2$ in the air (panel b) with respect to above canopy values, plotted against height above the forest floor (horizontal axis). Curve estimates are based on a negative exponential function, which resulted in the best fit to the data (i.e., highest R-squared). In cases where $CO_2$ concentration, or $\delta^{13}C$ were equal to above canopy values, we set the difference to 0.001 to estimates curves. Included data are from Sternberg *et al.* [3], Medina *et al.*[4], Broadmeadow *et al.*[5], Kruijt *et al.*[6], Lloyd *et al.*[7], Berry *et al.*[8], Buchmann *et al.*[9] [10, 11], and Hanba *et al.* [12], Ometto et al. [13], Berry et al.[8] Harwood et al.[14].

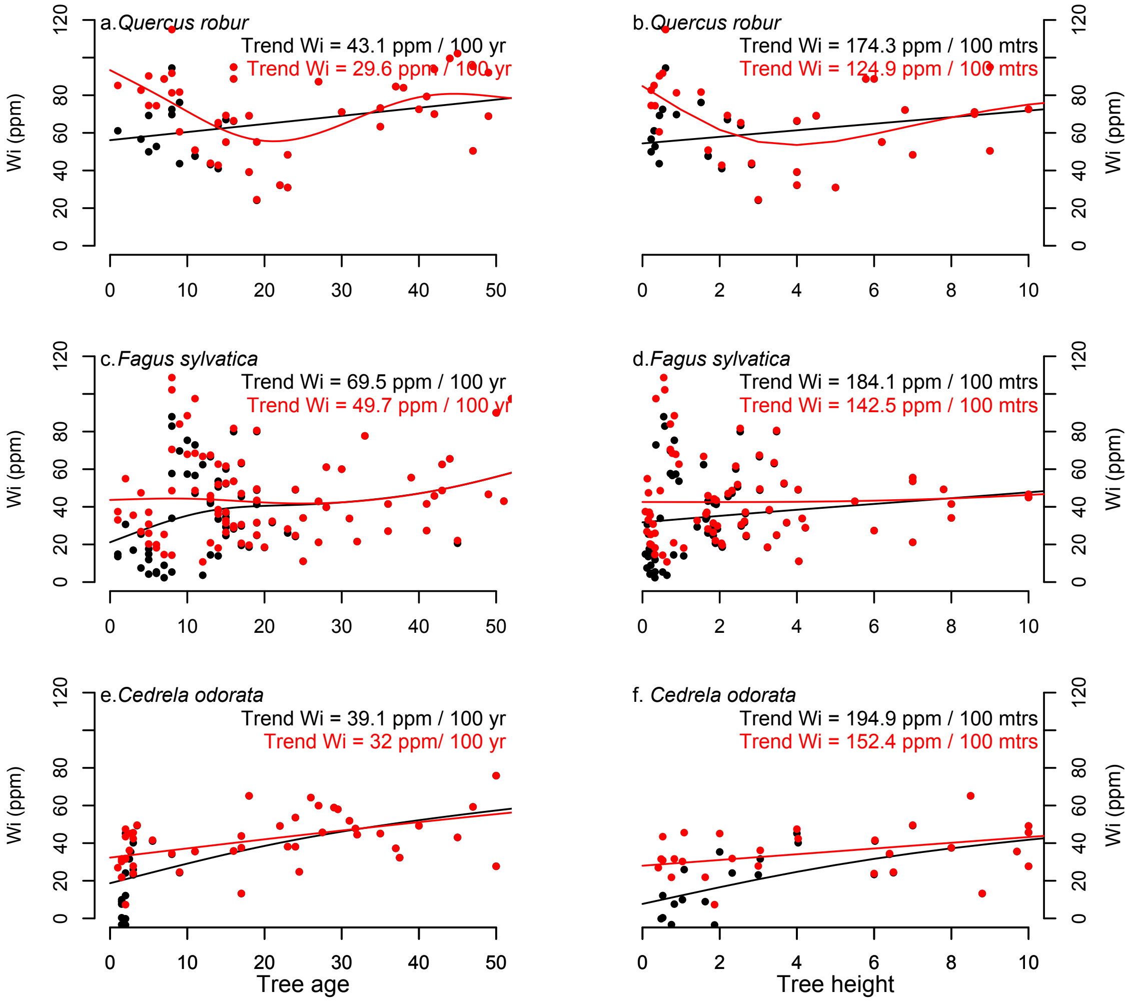


**Supplementary Figure 4. Original $W_i$ data and $W_i$ corrected for below-canopy differences in $CO_2$ concentration and $\delta^{13}C_{air}$.** Black points indicate original $W_i$ data, and red dots $W_i$ corrected for below-canopy differences in $CO_2$ concentration and $\delta^{13}C_{air}$ (see Methods). Graphs only show data for the first 50 years (panels a, c and e), and the first 10 meters of tree height increase (panels b, d and f) for the three broadleaf species. $W_i$ did not change in older and taller trees as below-canopy $CO_2$ concentration or changes in $\delta^{13}C$ are largely similar to levels above the canopy according to literature data (see SI Fig. 3). Note that we did not apply corrections for canopy $CO_2$ and $\delta^{13}C_{air}$ to the pine data as these trees grew predominantly in open conditions. Estimates of the trend were performed using General Additive Mixed Models (GAMM) from the gamm4 R package[2]. The values of the trend estimate are based on linear trend estimates for the full dataset, including older and taller trees (which are not shown in the graph).

**Supplementary Table 1. Overview of sample sites and species used in this study.** Upper part of the table provides details on new data collections for isotope analysis from sites in UK and North Bolivia, and information on the used sub-fossil *Pine* dataset from Helama et al.[1]. Lower section of table provides the sources of literature data on long-term trends in $W_i$ used for comparision, and displayed in main Fig.1 (colored lines).

**Newly collected data for this study**

| **Species** | **Sample sites (coordinates)** | **Number of trees** | **Mean temperature, rainfall** | **Vegetation type** | **Sampling strategy** |
|---|---|---|---|---|---|
| *Pinus sylvestris* | Loch-an-Eilein, Scotland, UK (57.13N, 3.83E), | 100 | 7.2°C, 977mm[1] | Temperate | Size stratified samples from extant living trees |
| | Lapland, Finland (68N, 23-28E)) | 180 | -2.4°C, 365mm[illegible] | Boreal forest and solitary pine trees | Subfossil ancient trunks, see Helama *et al.*[1] |
| *Quercus robur* | Bishop Wood, UK (53.79N, -1.15E) | 78 | 9.5°C, 626mm[1] | Temperate mixed forest | Size stratified samples from extant living trees |
| *Fagus sylvatica* | Bishop Wood, UK (53.79N, -1.15E), | 81 | 9.5°C, 626mm[1] | Temperate mixed forest, | Size stratified samples from extant living trees |
| | Leeds, UK (53.82N, -1.58E) | 33 | 9.5°C, 626mm[1] | Parkland and mixed forest | |
| *Cedrela odorata* | North Bolivia (-11.4, -68.72E -10.08N, -66.30E) | 75 | 27°C, 1700mm[3] | Tropical moist forest | Size stratified samples from extant living trees |

**Sourced literature data**

| **Species** | **Geographic area** | **Number of sites** | **Change in $W_i$ ppm $(100\ yr)^{-1}$ (from ~1900 to ~2000)** | **% change (from ~1900 to ~2000)** | **Reference** |
|---|---|---|---|---|---|
| *Pinus sylvestris* | Europe | 14 | 29[4] | 24%[6] | Saurer *et al.*[15], Frank *et al.*[16] |
| *Quercus robur* | Europe | 7 | 26[5] | 32%[6] | Saurer *et al.*[15], Frank *et al.*[16] |
| *Fagus sylvatica* | France | 2 | 23[4] | 22-44% | Duquesnay *et al.*[17] |
| *Fagus sylvatica* | Spain | 3 | 27 | 30-50% | Penuelas *et al*[18] |
| *Quercus robur* | UK, Finland | 5 | 26 | 35% | Waterhouse *et al.*[19] |
| *Fagus sylvatica* | UK | 1 | 42 | 48% | Waterhouse *et al.*[19] |
| *Pinus sylvestris* | UK | 1 | 40 | 40% | Waterhouse *et al.*[19] |
| *Cedrela odorata* | Brazil | 1 | 17[7] | 25%[7] | Hietz *et al.*[20] |

**References**

1. Helama S, Arppe L, Timonen M, Mielikäinen K, Oinonen M. Age-related trends in subfossil tree-ring δ 13 C data. *Chemical Geology* **416**, 28-35 (2015).

2. Wood S. gamm4: Generalized additive mixed models using mgcv and lme4. *R package version 01–2*, (2011).

3. Sternberg L, Mulkey S, Wright S. Ecological interpretation of leaf carbon isotope ratios: influence of respired carbon dioxide. *Ecology* **70**, 1317-1324 (1989).

4. Medina E, Sternberg L, Cuevas E. Vertical stratification of 13C values in closed natural and plantation forests in the Luquillo mountains, Puerto Rico. *Oecologia* **87**, 369-372 (1991).

5. Broadmeadow M, Griffiths H, Maxwell C, Borland A. The carbon isotope ratio of plant organic material reflects temporal and spatial variations in $CO_2$ within tropical forest formations in Trinidad. *Oecologia* **89**, 435-441 (1992).

6. Kruijt B*, et al.* Sources and sinks of CO2 in Rondonia tropical rainforest, inferred from concentrations and turbulence along a vertical gradient. In: *Amazonian Deforestation and Climate* (ed^(eds Gash J, Nobre C, J R, RL V). John Wiley (1996).

7. Lloyd J*, et al.* Vegetation effects on the isotopic composition of atmospheric CO2 at local and regional scales: Theoretical aspects and a comparison between rain forest in amazonia and a boreal forest in Siberia. *Aust J Plant Physiol* **23**, 371-399 (1996).

8. Berry SC, Varney GT, Flanagan LB. Leaf δ13C in *Pinus resinosa* trees and understory plants: variation associated with light and $CO_2$ gradients. *Oecologia* **109**, 499-506 (1997).

9. Buchmann N, Hinckley T, Ehleringer JR. Carbon isotope dynamics in Abies amabilis stands in the Cascades. *Canadian Journal of Forest Research* **28**, 808-819 (1998).

10. Buchmann N, Guehl JM, Barigah TS, Ehleringer JR. Interseasonal comparison of CO2 concentrations, isotopic composition, and carbon dynamics in an Amazonian rainforest (French Guiana). *Oecologia* **110**, 120-131 (1997).

11. Buchmann N, Kao WY, Ehleringer J. Influence of stand structure on carbon-13 of vegetation, soils, and canopy air within deciduous and evergreen forests in Utah, United States. *Oecologia* **110**, 109-119 (1997).

12. Hanba YT, Mori S, Lei TT, Koike T, Wada E. Variations in leaf β13C along a vertical profile of irradiance in a temperate Japanese forest. *Oecologia* **110**, 253-261 (1997).

13. Ometto JP, Flanagan LB, Martinelli LA, Moreira MZ, Higuchi N, Ehleringer JR. Carbon isotope discrimination in forest and pasture ecosystems of the Amazon Basin, Brazil. *Global Biogeochemical Cycles* **16**, (2002).

14. Harwood K, Gillon J, Roberts A, Griffiths H. Determinants of isotopic coupling of CO 2 and water vapour within a *Quercus petraea* forest canopy. *Oecologia* **119**, 109-119 (1999).

15. Saurer M*, et al.* Spatial variability and temporal trends in water‐use efficiency of European forests. *Global Change Biol* **20**, 3700-3712 (2014).

16. Frank D*, et al.* Water-use efficiency and transpiration across European forests during the Anthropocene. *Nature climate change* **5**, 579-583 (2015).

17. Duquesnay A, Breda N, Stievenard M, Dupouey JL. Changes of tree-ring delta C-13 and water-use efficiency of beech (Fagus sylvatica L.) in north-eastern France during the past century. *Plant Cell and Environment* **21**, 565-572 (1998).

18. Penuelas J, Hunt JM, Ogaya R, Jump AS. Twentieth century changes of tree‐ring $\delta$ 13C at the southern range‐edge of *Fagus sylvatica*: increasing water‐use efficiency does not avoid the growth decline induced by warming at low altitudes. *Global Change Biol* **14**, 1076-1088 (2008).

19. Waterhouse JS*, et al.* Northern European trees show a progressively diminishing response to increasing atmospheric carbon dioxide concentrations. *Quaternary Science Reviews* **23**, 803-810 (2004).

20. Hietz P, Wanek W, Dunisch O. Long-term trends in cellulose delta C-13 and water-use efficiency of tropical *Cedrela* and *Swietenia* from Brazil. *Tree Physiology* **25**, 745-752 (2005).
